# Dental Society of Western New York

**Published:** 1867-11

**Authors:** R. G. Snow


### DENTAL REGISTER.

VOL. XXI.] NOVEMBER, 1867. [No. 11.

### Original Communications.

### DENTAL SOCIETY OF WESTERN NEW-YORK.

#### RETIRING ADDRESS OF THE PRESIDENT, R. G. SNOW.

**Read before the Dental Society of Western New York, October 1, 1867.**

GENTLEMEN :—The time having now expired that, by your partiality, I was called to preside over your deliberations, custom has made it incumbent on me to read you a short address. And, in endeavoring to discharge this duty, I cannot hope to advance anything new, and I fear nothing that will even interest you. The choice of a subject is a matter of no little embarrassment, and after some reflection I have thought that a slight and rapid review of our profession and some of its improvements would be as acceptable as anything I can offer you.

There can be little doubt that the inventive genius of any people, and even the desire to adopt improvements when made, is greatly influenced by the Government under which they live, and the amount of liberty which they enjoy.

As an example, compare our own and the European nations, and I think you will agree with me, that where the greatest liberty is enjoyed by the people, there will be found the greatest advancement in the arts and sciences, and the greatest perfection in everything which adds to the enjoyment and happiness of man.

This is rendered still more apparent by comparison with any of the Asiatic nations, most, if not all, of whom live under a despotic form of Government, the people being perfectly satisfied to live and do just as their forefathers did two and three thousand years ago. I lately conversed with a missionary who had spent some fifteen years in Persia, and his account of his experience there would seem to indicate that the task of inducing the people to change their habits and improve their condition was quite hopeless. They (the Missionaries) had taken out plows, and various agricultural implements, in hopes of inducing them to improve their mode of agriculture, but the inhabitants would not adopt them. Their fathers got along very well without anything of the kind, and they were entirely satisfied to do the same. The spirit of improvement is entirely wanting among them. Their Dentistry consists entirely in extracting the teeth when they are diseased and painful.

Liberty and improvement seem to go hand in hand; and this idea must have been uppermost in the mind of our illustrious countryman, Daniel Webster, when he exclaimed in one of his speeches, "I thank God I was born in this land, and in this age; for this is the land of liberty, and this is the age of improvement." Perhaps, gentlemen, we can all endorse this sentiment.

Some wise man has said that there is always a best way of doing anything. I do not think this proposition will be disputed by any one, and we Americans, as a people, seem to have fully adopted the idea, and are constantly engaged in every branch of the mechanic arts, in contriving and inventing instruments and machinery for doing things quicker, cheaper, better, and in many things the degree of perfection arrived at is truly surprising. From the making of a pin or a carpet tack to the most massive steam engine, everything is turned out in great perfection, and with astonishing rapidity. And thus we now have improved methods of building and warming our houses—improved methods of traveling—improved methods of sending intelligence from one point to another—improvements in agriculture—in printing—in short, in every department of knowledge; and last, though perhaps not least, improvements in Dentistry. The inventive genius of our people was probably never more active than now. The records of the patent office in Washington show that from two to three hundred patents are issued every week—two hundred and sixty being about the average.

But, not to pursue this train of thought any further at present, let us turn our attention more directly to our own profession. And now I may say, that the improvements in any branch of knowledge can only be fully understood and appreciated by comparing the dim, distant past with the present.

Among the ancients the science and art of Dentistry was undoubtedly in a very rude and uncultivated state, though the Greeks and Romans, and Egyptians are said to have carried it to some degree of perfection.

The first account which we have of any attention being paid by mankind to their teeth is from the Greek historian Hero [illegible]itus, who lived about three hundred years before the Christian era. He states that the Egyptians at a very remote period confided their teeth to the care of a particular set of persons, but he does not state in what this care consisted.

Little, however, is known of the attainments of these early practitioners. In the ancient tombs of this people, it is stated that artificial teeth of ivory or wood have been found, some of which were fastened on gold plates. It is also stated that the teeth of mummies have been found filled with gold. Thus it would seem that the ancient Egyptians understood processes of the art, which are commonly regarded only as the inventions of the refined nations of modern times.

Hippocrates, also a Greek, who lived about the same time, said to be the first correct observer, and the first good physician the world ever saw, gives a very amusing if not ludicrous account of the origin and development of the teeth. He says, "there is a glutinous increment from the bones of the head and jaws, of which the fatty part is dried by heat, and burned up—and the teeth are made harder than the other bones, because there is nothing cold in them." He also describes some of the diseases of the teeth, and recommends their being removed when they are rotten and loose. He, however, describes no instruments for this purpose.

He gives the following receipt for a dentifrice: "take the head of a hare and three mice, burn and reduce them to powder, then mix them with an equal weight of powdered marble." Here we have animal charcoal and prepared chalk, though in rather a crude state.

Martial, a Greek poet, addressing himself to his friend Selius, says, "you are not ashamed to buy teeth and hair, but what will you do for an eye, as there are none to sell?"

This would seem to indicate that among the Greeks, at least, their Dentists were sufficiently skilled to insert artificial teeth. It would appear, however, that they were made of bone and ivory, as it is stated that the toothless mouth of Egel was repaired with bone and ivory.

Celsus, a Roman physician, gives the first regular directions for extracting the teeth. His method was to imitate the natural loosening of the teeth by shaking them well in the jaw, and subsequently extracting them. The severity of the operation, as practiced by him and others in his time, may be imagined, when it is stated that he preferred the application of the hot iron or boiling oil to the tooth to make it exfoliate.

With the decline and fall of the Greek and Roman Empires, Dentistry fell into disuse, or was practiced by empirics and pretenders, and as late as 1728 when Fauchard published his work on the structure and diseases of the teeth, in speaking of the state of Dental surgery in his time, remarks, "that in France, the most celebrated surgeons having abandoned this branch of surgery, or having but little cultivated it, their negligence gave rise to a class of persons who, without theoretic knowledge or experience, and without being qualified, practiced it at hazard, having neither principles nor system. About this time, however, it was provided by law, in France, that those intending to practice Dental surgery should submit to an examination by men learned in all the branches of medical science who should decide upon their merit.

Ambrose Paré, a French anatomist and surgeon, in 1579, gives a very good account of the teeth, and says that their adherence to the jaw is caused by a ligament which goes from the root of the tooth to the jaw. He also thought that the teeth continue to grow during the life time of the individual, and that they can distinguish tastes. He cured toothache by the hot wire, and also by filling the tooth with cotton wet with sulphuric or nitric acid.

This ligament mentioned by Ambrose Paré, must be the same that Dr. Caldwell, a Dentist of Philadelphia, made so much noise about some twenty years ago. He (Caldwell) was in the habit of cutting it with a lancet, thereby, as he pretended, rendering extraction very easy and almost painless. I have heard nothing of this ligament since Dr. Caldwell's time.

The following case is related by Ambrose Paré which shows that the operation of transplanting the teeth was in vogue in the 16th century :

"A princess was obliged to have a diseased tooth extracted, and on applying to a surgeon she brought with her one of her waiting maids, who stood by the side of her mistress, and when the tooth of the princess had been extracted, a sound one was taken from the jaw of the girl, and placed into the socket of the jaw of the former, which took root and became sound, healthy and useful."

Senwenhoeck, a Hollander, in his investigations with the microscope, in 1678, discovered the tubular structure of the teeth, and published the same in the English philosophical transactions of the same year. He says, that he and another gentleman plainly saw that the whole tooth was made up of very small, straight and transparent pipes—six or seven hundred of the pipes put together he judged not to exceed the thickness of one hair of a man's beard. His statements, however, were so new and extraordinary, that but little attention was paid to them at the time—though his account of the structure of the teeth is now recognized as in the main correct. He was a hundred years or more in advance of his age, and was therefore not fully appreciated during his lifetime.

In England, also, Dentistry remained in a very uncultivated state; and down to the time of John Hunter, little or nothing had been done to raise it to the dignity of a science. His work on the natural history of the teeth he published in 1771, and that on the diseases of the teeth, and their treatment, in 1778. And although his views were greatly in advance of any previous English writer on the subject, still they were different in many respects from those held at the present day. He instituted a series of experiments to test the question whether the teeth were vascular or were endowed with vessels and a circulation like the other parts of the body, and his observations and experiments led him to the belief that they were not—that they had no vessels and no circulation.

These are his words after relating his experiments: "It would appear then that the teeth are to be considered as extraneous bodies with respect to a circulation through their substance—but they have most certainly a living principle by which means they make part of the body, and are capable of uniting with any part of a living body."

Perhaps I may be allowed to relate to you one; and probably the most convincing to his mind of any of his experiments.

It is known that by feeding a young animal with food mixed with madder, the bones become tinged a red color. Mr. Hunter failing in his efforts to inject the teeth as he was able to the other bones, made this experiment.

He shut up several pigs and fed them three or four weeks on food mixed with madder. He then killed one of them and found the bones tinged red. But the parts of the teeth formed before the feeding were white as before, while the layer of tooth formed during the feeding was tinged red. He then fed them with food without the madder for about the same length of time. He then found the bones assuming their natural white color; but the red layer remained in the teeth the same as before, with the addition of a white layer outside the red. And he argues from this, that by feeding a young animal with and without madder for a month at a time, alternately, when the teeth are forming, you will have alternate layers of red and white through the whole tooth; and the fact that this coloring matter, after being deposited in the teeth, remained there permanently, was satisfactory proof to his mind that the teeth contained no absorbent vessels. How long after feeding these animals with madder he made an examination of their teeth he does not say. It would have been much more satisfactory if he had, for although the coloring matter might not have been removed in a month or two, it might have been in a year or two.

But these views of Mr. Hunter are now considered by all well informed Dentists to be erroneous. Mr. Thomas Bell, of London, in his work on the teeth, speaking of the difficulty of injecting them, says, "what art has failed to do, nature or rather disease has done for us—and then goes on to say that he has in his possession a set of teeth from the mouth of a young woman, who died of jaundice, every tooth of which is tinged a bright yellow color. Authors, also, tell us that the teeth of persons who die of strangulation, as hanging or drowning, are very commonly more or less injected with red blood; also, in some inflammatory diseases. And, more than all, every Dentist has had more or less trouble with sensitive dentine, and sensitive dentine would seem to indicate both vessels and nerves.

Mr. Hunter's views in regard to the diseases of the teeth were in the main correct, but his directions in regard to Dental practice, show plainly enough that he was not a practical Dentist; and can hardly fail to excite a smile when recapitulated at the present day. For instance, when a molar tooth is decayed in such a place that it cannot be filled—or when it is decayed to the nerve, and still enough of the tooth left to be useful, he recommends that it be extracted and boiled in water a few minutes to make it clean and destroy its life, and then returned to its socket in the jaw. This, he says, will prevent any further decay as the tooth is now dead, and not to be acted on by any disease, but can only suffer chemically and mechanically. He adds, that this practice has been sometimes followed with success

He mentions two metals only for filling decayed teeth, gold and lead; and says, when a large cavity is filled in the crown of a molar, for instance, it is well to bore a small hole through the wall of the tooth, and put in a pin to prevent the filling from coming out.

Now, all this sounds very strangely at this day, but it was certainly recommended by good authority, and was undoubtedly thought by many to be good practice, less than a hundred years ago.

Mr. Hunter also taught and practiced the operation of transplanting the teeth from the mouth of one patient to that of another, but as diseases were sometimes communicated in this way, it soon fell into disuse.

But, gentlemen, let us come down to our own times and our own country—and now, truth compels me to say that up to within the last forty or fifty years our profession was in a very neglected and uncultivated state. With some few honorable exceptions in our larger cities the practice was in the hands of adventurers and charlatans. It was practiced by barbers, at least so far as extraction is concerned. I think it may admit of some doubt whether our profession fifty years ago was in a greater state of advancement in this country, than it was two and three thousand years ago among the Greeks and Romans and Egyptians. And considering how it was treated, it can hardly seem strange that it was so. Our medical colleges were silent on the subject of the diseases of the teeth. None of our eminent Surgeons cultivated this branch of surgery, and Dentistry seemed to be left to take care of itself; or to fall into the hands of any one who had the enterprise and ingenuity to contrive up a kit of tools and proclaim himself a Dentist. I do not think that I am far from the truth when I say that it became a kind of refuge for persons who were broken down in other kinds of business. Hence, the majority of itinerant Dentists that used to perambulate the country.

Under these circumstances Dentistry and Dentists must and did, sink in the public estimation to the very lowest ebb. Dr. Horace H. Hayden, of Baltimore, one of the most celebrated Dentists of his time, in his remarks before the first Dental convention ever held in this country, said among other things, that when he entered on the duties of the profession, forty-three years before that time, the very name of Dentist was a reproach and a bye word, and this condition of things continued for many years after that time.

But I am happy in saying that a great change for the better has come over the members of our profession. That spirit of narrow-mindedness and illiberality formerly existing, has, in a great measure disappeared, and the avenues to knowledge are now open and free to all ; and the interchange of ideas and sentiments among Dentists almost universal. Dentistry has been liberalized and raised in the public estimation, and its sphere of usefulness greatly extended. I attribute these changes to three causes. The publication of Dental journals ; the establishment of Dental colleges, and the formation of Dental associations.

The first number of the *American Journal of Dental Science* was issued in the fall of 1839, under the auspices of a committee of liberal minded gentlemen. Its first editors were Eleazer Parmley, of New York, and Chapin A. Harris, of Baltimore. The object of its publication as set forth in the prospectus, was to make it a vehicle of useful information to the numerous Dentists in the United States. This, so far as I know, was the first Journal ever published, devoted exclusively to Dental science.

In 1840, the first Dental college in the United States, and probably the first in the world, was established in Baltimore, and to Chapin A. Harris, in a great measure, is due the honor of inaugurating this important movement. The first course of lectures was given in the fall and winter of 1840 and 1841.

The first professors were Horace H. Hayden, H. Willis Baxly, Chapin A. Harris, Thomas E. Bond, Jr.

Only four, as you perceive. Now the same college has eight professors and a demonstrator. At the close of the first course of lectures two students graduated and received the title of D. D. S. The next year three graduated.

On the 18th day of August, 1840, the first Dental convention commenced its session in New York. A constitution was adopted and officers chosen. Fifteen Dentists signed the constitution and by-laws at this, the first session of the convention. This was twenty-seven years ago. Now, we have in the United States no less than six Dental colleges; we have four ably conducted Dental journals, and Dental societies almost everywhere.

But I wish to direct your attention, for a moment, to some of the improvements which have been introduced into our profession within the last twenty or thirty years. It is curious to observe how gradually, step by step these improved methods of practice have advanced to their present state of perfection.

And first, of the operation of filling. The directions given by the older authors, for performing this operation are not in general sufficiently specific. Dr. Fitch directs, after the cavity is properly prepared and cleansed, and dried, with a blunt pointed instrument, to pass in the gold leaf until the cavity is full, and press it down very hard. He gives no directions as to the manner of preparing the foil, or the manner of introducing it into the cavity, leaving that entirely to the skill of the operator, and every operator to adopt his own method.

Dr. Harris is more specific and directs the gold to be cut into strips and rolled or twisted into a rope—then, after the cavity is prepared, with due precaution as to keeping it dry, set the end of the rope to the bottom of the cavity and with an instrument fold it down, and carry the fold to bottom of the cavity so that the end shall present to the surface, and so on till the cavity is filled. This method was almost universally in use, when I came into the profession twenty-eight years ago. Harris advocates wedge shaped instruments, and directs them to be left rough on their sides and points so as to hold the gold.

But the idea of having the points serrated does not seem at that early day, to have fully dawned upon the profession.

Then the idea of folding the foil into a ribbon or tape and introducing in the same manner, was adopted by many Dentists. This was held to be an improvement, as the different layers of foil would lie flat upon each other and against the walls of the cavity, and therefore make a more compact filling than when used as a rope.

Then the plan of rolling up the tape into a hard coil, or cylinder part of the way—set this coil into the cavity and fold in the balance of the tape in the usual way.

Then the method of rolling up a series of cylinders and setting them into the cavity, like cigars in a tumbler, forcing them against the walls of the cavity, and introducing smaller ones till it is completely filled. This method, I believe was introduced to the profession by Dr. Clark, of New Orleans.

But what has produced the greatest revolution and improvement in our manner of filling, has been the introduction of adhesive gold, serrated instruments, and the mallet. With these the skillful operator is able not only to fill a cavity and restore the tooth to health and usefulness, but to build out and restore the shape of a broken tooth, or if necessary build on an artificial crown. This, I think, may be looked upon as the perfection of this difficult and most useful operation.

The treatment of teeth when decayed so as to expose the nerve, has, within the last few years undergone great and important improvements. Previous to 1835 or '6, when Dr. Spooner promulgated to the profession the fact that arsenic would destroy the vitality of the nerve, there was no settled treatment for teeth in this condition. Every Dentist, and I may say every writer on Dentistry, had his own particular method; and the result was in a great majority of cases, that the treatment ended in extraction. Indeed, most authors up to that time, and even later recommended the molar teeth to be extracted when decayed to the nerve, looking upon all curative treatment as useless and hopeless.

Dr. Kœcker recommended the exposed point of the nerve to be seared with a red hot blunt pointed instrument, taking great care not to destroy the entire pulp, then lay over it a small piece of sheet lead and fill the tooth. He claims to have practiced this method successfully.

Dr. Fitch has several methods: destroying the nerve with some of the mineral acids; or lunar caustic; or in the single teeth with the red hot wire—applying powdered nutgalls to the exposed nerve for a month or more and eventually capping with lead or gold and filling. But he closes by saying that many teeth will resist all treatment and must be extracted.

Mr. Fox speaks of luxating the tooth in its socket so as to break the nerve and bloodvessels at the point of the fang, and let the tooth remain. This was soon abandoned as being no better than Mr. Hunter's method already mentioned.

A Mr. Fay, of London, published a paper as long ago as 1830, in which he advocated the plan of cutting off the tooth close down to the gum—and this method was, for a time, quite popular in Europe—I am not aware that it was ever practiced in this country. But it was soon found that the fangs were nearly as painful as before the excision, and it soon fell into disuse. Other plans have been proposed, but I will not trouble you with them.

I have said that previous to the advent of arsenic there was no settled method of treating exposed nerves. There was not for many years after, and I can hardly say that there is now. Every Dentist knew that arsenic would destroy the life of the nerve, but no one yet knew how to treat a tooth after the nerve was dead to restore it to health and usefulness. Harris, as late as 1845, speaks very disparagingly of its use, and says the teeth are rendered almost useless, and if filled after the nerve is destroyed in this manner alveolar abscess is almost sure to follow. To prevent the formation of the abscess, some one proposed to insert a piece of hollow gold wire or tube either through the filling, or through the wall of the tooth to communicate with the nerve cavity and allow the escape of offensive secretions. This was soon simplified by drilling a small hole through the wall of the tooth under the gum. But it was found that this allowed the decay to go on in the centre of the tooth so that it was soon destroyed. And now came the last and certainly a very important step in this improved manner of treating nerve cases—an idea which it took the profession twenty years or more to arrive at, after the advent of arsenic—the idea of cleaning out the nerve cavity and after proper treatment filling the fang to the apex with metal. This when properly done is almost uniformly successful.

One thing more, and I will pass from the nerves. In 18[illegible]9, Dr. E. Baker, of New York, in an article in the Journal of Dental science, recommended the nerves in the front teeth and even the bicuspids when exposed to be removed by a small instrument or broach passed up to the end of the fang so as to bring it out at once. Then the whole internal cavity wiped dry and immediately filled with gold to its highest point. He says there is seldom any inflammation following the operation, or any unpleasant symptom whatever. He also says that Dr. Hudson, of Philadelphia, followed this practice more than thirty years before the time in which he (Baker) wrote and claims that teeth filled by Hudson thirty years before, were still good and serviceable. I do not think that this practice ever came into general use in the profession; but I am happy to say that it is being revived by some of our members.

The improvements in this branch of practice may be looked upon as important as any which have been introduced into our profession for many years past; as the skillful operator is now enabled to preserve and make useful for an indefinite length of time, many teeth which formerly were doomed to extraction.

The improvement in artificial teeth within the last forty years has been most remarkable. Forty years ago artificial substitutes for the natural teeth were made almost entirely of perishable materials, such as bone, ivory, cattle's teeth or human teeth. Now the porcelain teeth offered in market are strong, imperishable, and very perfect imitations of the natural organs. The manufacture of porcelain teeth seems to have been a French invention; but to our American Dentists is due the credit of bringing them to their present state of perfection.

The first experiments in the manufacture of these teeth in the United States, was made as early as the year 1807 by Mr. Charles W. Peale, of Philadelphia. He had the misfortune to lose a number of his teeth and had some made of ivory. Seeing an account in the papers of mineral teeth, he procured a receipt and materials and commenced experimenting, and soon produced so good an article that he constructed a set on gold plate for his own use, and also made several sets for his friends. He also gave instructions to Dr. Barabine a Dentist then practicing in Philadelphia.

This Mr. Peale seems to have been a kind of universal genius. In his boyhood he was apprenticed to a saddler, and in after life successively carried on the business of saddle and harness making, silversmith, watchmaker, carver, became an eminent portrait painter—a sportsman—a naturalist—a taxidermist—founded the Philadelphia museum—made himself a violin and a guitar—and invented and constructed several machines. It can hardly be looked upon as singular that a man who could do all this, should be able to make porcelain teeth.

It is stated that the first regular manufacturers were Greenwood, Hoffendale & Parkhurst, who were engaged in the business about the year 1825. French artificial teeth were manufactured in Philadelphia from 1827 to 1830, by Planteau and McHenry. Up to this time it does not seem that any effort had been made to bring these teeth before the profession so as to introduce them into general use. The probability is that all the teeth made up to this time were of so inferior a quality as to be unsaleable. About the year 1835, Samuel W. Stockton, of Philadelphia, produced a very excellent and natural looking tooth and manufactured them in large quantities—threw them into market, and they were very soon universally adopted by the profession. Since that time various persons have engaged in the business, and by gradual improvements have brought them to their present state of perfection.

The manner of fastening artificial teeth in the mouth has also greatly improved within the last few years. It is stated that as long ago as three or four hundred years before the Christian era, partial sets of teeth were held in the mouth by being fastened to the remaining teeth by ligatures of silk or flax or wire of silver or gold; and this method was still in use fifty years ago, and continued in use by many Dentists nearly if not quite down to the advent of porcelain teeth. Then the clasp came into use and was certainly a great improvement over ligatures or wires, as it enabled the wearer to take out and replace his teeth at pleasure and without difficulty. Mr. James Gardette seems to have been one of the first to use clasps. He settled in Philadelphia in 1784, and continued in uninterrupted practice until 1830, a period of forty-six years. He was the first also to put in whole sets of teeth on the principle of atmospheric pressure. One would suppose that this method would suggest itself to the mind of any person who had any knowledge of natural philosophy. But its discovery by Mr. Gardette, was quite accidental. You are all aware that the old method was to support them by spiral springs, and it was supposed that they could not be worn without the springs. About the year 1800 he made a set of upper teeth for a lady of the hippopotamus ivory, and attached the springs in the usual way. They soon decayed and became discolored and a second set became necessary. At the appointed time the lady came for her teeth—they were finished but the springs were not attached. He told her to take them home and keep them in her mouth as much of the time as she could, so as to become used to them, and he would call in a few days and attach the springs. Happening to be very busy he neglected to call for some three months; when with springs and pliers and many apologies for not calling sooner, he confronted his patient and asked for the teeth for the purpose of putting on the springs, and was greatly astonished when told that with the exception of the first few days, she had worn them quite as comfortably without the springs as she had the others with. This was an entirely new idea to Mr. Gardette. He at once comprehended the philosophy of it, and seems to have profited by it afterwards by adopting it in his practice. But it does not appear that this method came to be generally known, and practiced by the profession for more than thirty years afterwards. It is not probable that Mr. Gardette took any pains to communicate it to his professional brethren. That was not the custom in those days. In the second edition of Dr. Fitch's work published in 1835, it is not even mentioned. In the edition of Harris, published in 1845, he describes, minutely, the method of making and applying spiral springs and concludes the chapter by saying that in some cases, by making the plate wider and getting a very accurate fit, sets can be worn without the springs. He speaks approvingly of the method—says it is preferable to any other. He makes no mention of the air chamber which has been so universally in use for many years past. I am not aware that spiral springs are in use by any one at the present day.

Many other improvements might be mentioned—the improved form of instruments, and all the necessary appliances of our profession now so easily obtained at the Dental depots—but I have already trespassed too long on your time and patience. But before closing, allow me to ask you to compare for a moment the condition of the Dental profession now, with what it was two thousand years ago—what it was a hundred years ago—what it was thirty years ago—and you will hardly fail to form some idea of the great and wonderful progress made. It is less than thirty years since the formation of the first Dental society in this country; but it is not to be supposed that it was formed without opposition. Many Dentists scouted the very idea, and would have nothing to do with it; and it is to the energy and perseverance of those high toned and liberal minded gentlemen who inaugurated the important movement of establishing Dental Journals, Dental Colleges and Dental Societies that we are indebted for these great results.

Dental Societies have had, and are having very great influence in improving our practice—in elevating our profession, and making it more respected by the community.

I know there are Dentists even now, who do not approve, or at least who do not attend the meetings of any society. But I venture to say that they are men who are satisfied with moderate attainments, and in whom the spirit of improvement does not exist. They have fallen into a kind of routine practice and are satisfied to remain so. But the most eminent practitioners of our art, the shining lights of our profession, are to a man in favor of societies.

Those who opposed societies were in favor of elevating the dignity and extending the usefulness of the profession, but they proposed to do it by individual effort—which means, so far as I have been able to ascertain, to keep all the knowledge you have, and get all you can, but never communicate t to your neighbor.

I venture to affirm that more progress has been made in the last thirty years by the combined agencies which I have mentioned, than would have been accomplished in a thousand years by individual effort.

Individual effort means—let every man take care of himself—and others take care of themselves—it means elevate yourself, but keep down your neighbor—in one word it means selfishness.

Gentlemen, if you drop a pebble into a pond of water you immediately see a circling ripple widening and spreading till it reaches the outmost bounds of the pond; and strictly speaking it may be said that every particle of water in the pond has been influenced and moved by the pebble. If instead, you put the pebble in your pocket no such result is produced.

If you drop a new idea in this or any other society, in like manner a circling ripple is formed and spreads from mind to mind till it permeates, influences, moves, and improves the entire body of the profession. If, instead, you keep it to yourself, and use it merely for your own benefit, no such result is produced.

Let me say then, in conclusion, that free discussion—the free interchange of ideas, and combined effort, are the true means of progress and advancement.

And now, gentlemen, in retiring form this chair, allow me to thank you for the confidence reposed in me—and for the uniform kindness always manifested towards me—and to assure you that my sympathies and best wishes are with you in everything that tends to promote the interests of our Association, or to the improvement of our noble profession.

